# Neuromorphic Photonics Circuits: Contemporary Review

**DOI:** 10.3390/nano13243139

**Published:** 2023-12-14

**Authors:** Ruslan V. Kutluyarov, Aida G. Zakoyan, Grigory S. Voronkov, Elizaveta P. Grakhova, Muhammad A. Butt

**Affiliations:** 1 School of Photonics Engineering and Research Advances (SPhERA), Ufa University of Science and Technology, 32, Z. Validi St., 450076 Ufa, Russia; 2 Samara National Research University, 443086 Samara, Russia

**Keywords:** neuromorphic computing, photonic integrated circuit, imaging, artificial intelligence, machine learning

## Abstract

Neuromorphic photonics is a cutting-edge fusion of neuroscience-inspired computing and photonics technology to overcome the constraints of conventional computing architectures. Its significance lies in the potential to transform information processing by mimicking the parallelism and efficiency of the human brain. Using optics and photonics principles, neuromorphic devices can execute intricate computations swiftly and with impressive energy efficiency. This innovation holds promise for advancing artificial intelligence and machine learning while addressing the limitations of traditional silicon-based computing. Neuromorphic photonics could herald a new era of computing that is more potent and draws inspiration from cognitive processes, leading to advancements in robotics, pattern recognition, and advanced data processing. This paper reviews the recent developments in neuromorphic photonic integrated circuits, applications, and current challenges.

## 1. Introduction

Neuromorphic photonics represents a cutting-edge, multidisciplinary realm at the confluence of artificial intelligence (AI), photonics, and neuroscience [1]. Its overarching goal is nothing short of a transformative evolution in computing, seamlessly uniting the foundational principles of neuromorphic computing with the swiftness and efficiency inherent in photonics [2]. This inventive paradigm employs light-based neurons and optical synapses to emulate the intricate behaviors of human brain cells closely, resulting in specialized hardware uniquely tailored for the domains of AI and machine learning [3]. The standout feature of this field is its remarkable energy efficiency, enabling lightning-fast, parallel data processing while conserving power resources. By harnessing the velocity of light and mirroring the intricate neural networks (NNs) of the human brain, neuromorphic photonics has the potential to unlock entirely novel horizons in high-performance computing, poised to dramatically elevate applications in pattern recognition, data manipulation, and intricate problem-solving [4,5]. While still in its infancy, this field holds promise of more capable and efficient AI systems, with the potential to fundamentally reshape the computing landscape [6].

AI technologies, encompassing facial recognition, machine learning, and autonomous driving, are reshaping our daily lives [7,8]. Deploying of task-specific AI systems demands training NNs with extensive datasets on conventional computers. However, limitations in throughput and efficiency due to prevailing computer architectures currently hinder this process [9]. Drawing inspiration from the intricate architecture of the human brain, researchers are pioneering the development of next-generation intelligent computing systems designed to emulate synapses and neurons. These systems encode information using spatiotemporal pulse patterns generated by presynaptic neurons, with postsynaptic neurons accumulating and generating new neuronal pulses upon reaching stimulation thresholds. By integrating myriad neurons, these systems give rise to nonlinear spiking NNs, enabling information processing through spatiotemporally encoded neuron pulses. Intel’s TrueNorth chips, for instance, have achieved a remarkable level of energy efficiency, surpassing conventional microelectronic chips for specific AI tasks and rivaling the computational capabilities of the human brain [10]. Nevertheless, the scalability of integrated neurons remains hampered by challenges such as electrical interconnect bandwidth, pulse loss, and communication delays. Optical interconnects, offering substantial bandwidth, minimal loss, and negligible latency, have the potential to address these electrical interconnect limitations [11].

The demands of real-time, data-intensive, intelligent information processing tasks underscore the need for innovative and smart optimization hardware. Convolutional neural networks (CNNs) excel at extracting hierarchical feature maps to enhance recognition accuracy, and there is a growing interest in employing photonics for their implementation. In this context, a large-scale and adaptable photonic convolutional neural network (PCNN) that leverages a hardware-friendly distributed feedback laser diode (DFB-LD) is proposed [12]. This approach involves applying a biological time-to-first-spike coding method to a DFB-LD neuron to execute temporal convolutional operations (TCO) for image processing. In practical experiments, PCNN successfully employs TCO to extract image features using 11 × 11 convolutional kernels. Additionally, the temporal pulse shaping of a DFB-LD neuron is explored to construct a densely connected and fully connected layer, enabling rapid adjustments of synaptic weights at a remarkable rate of 5 GHz and providing high classification accuracy in benchmark image classification tasks, with 98.56% for MNIST and 87.48% for Fashion-MNIST. These findings underscore the potential of optical analog computing platforms resembling neurons for real-time and intricate intelligent processing networks [13].

This paper is meticulously organized as follows: Section 2 provides a comprehensive overview of the current market size of neuromorphic computing. Section 3 delves into the intricacies of neuromorphic photonic integrated circuits, encompassing topics such as deep neural networks, neural networks involving complex arithmetic calculations, spike neural networks, convolutional neural networks, methodologies for implementing activation functions in optical neural networks, and programmable photonic neural networks. Moving on to Section 4, we spotlight the most significant applications of neuromorphic photonics, embracing areas like neuromorphic computing-based photonic integrated circuits, neuromorphic imaging, and image processing via neuromorphic structures. Section 5 is dedicated to thoroughly discussing the prevailing challenges of developing neuromorphic photonics. Finally, in Section 6, the paper concludes with insightful remarks summarizing key findings and contributions. This deliberate structure ensures a systematic exploration of the diverse facets of neuromorphic photonics, from market dimensions to cutting-edge applications and challenges, providing a comprehensive understanding for readers.

## 2. Neuromorphic Computing Market Size

**Figure 1:**
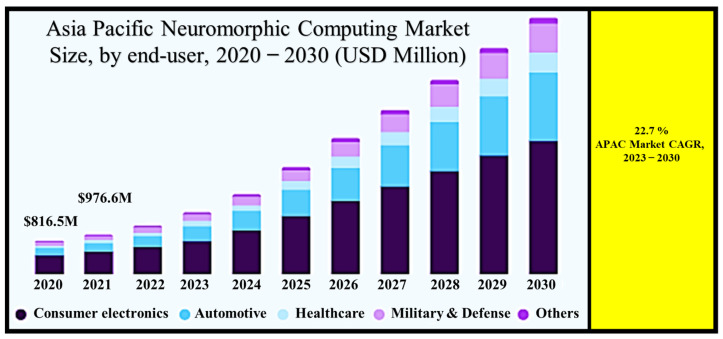
Neuromorphic computing market. Inspired by [14].

The global neuromorphic computing market reached an impressive value of USD 4237.7 million in 2022, and its trajectory is nothing short of remarkable. Projections indicate that this market is set to undergo a staggering expansion, with a projected compound annual growth rate (CAGR) of 21.2% from 2023 to 2030 [14], as shown in Figure 1. What fuels this extraordinary growth? It is the ever-increasing adoption of neuromorphic technology across a broad spectrum of applications. Notably, integrating neuromorphic technology in deep learning (DL) applications, transistors, accelerators, next-generation semiconductors, and autonomous systems, such as robotics, drones, self-driving cars, and artificial intelligence, are key drivers behind this surge. For instance, in August 2022, a trailblazing multidisciplinary research team achieved a breakthrough with NeuRRAM, a cutting-edge neuromorphic chip. This innovative development promises to revolutionize AI applications by delivering superior accuracy while consuming significantly less energy than other platforms. As we move forward, the global neuromorphic computing market is poised to play a pivotal role in shaping the future of technology.

In the realm of neuromorphic computing, the power of intricate algorithms lies in their ability to efficiently execute within robotic systems, offering an impressive blend of superior performance and minimized energy consumption. This capability is a cornerstone for creating cutting-edge robotic systems that operate with remarkable efficiency and precision. Illustrating the potential of this technology, in September 2022, Intel Corporation embarked on a groundbreaking collaboration with the Italian Institute of Technology and the Technical University of Munich. Their joint endeavor introduced a novel object-learning method deeply rooted in neural networks (NNs). This collaborative partnership is dedicated to harnessing the prowess of neuromorphic computing using an interactive online object-learning approach. The goal is to empower robots to learn about new objects swiftly and accurately post-deployment, enhancing their adaptability and capabilities.

Furthermore, leading companies in the market are actively investing in continuous research and development initiatives while introducing innovative products that drive the frontiers of research technology. An illustrative example comes from December 2022 when Polyn Technology, an Israel-based Fabless semiconductor company, made a significant announcement [15]. They unveiled the availability of neuromorphic analog signal processing models designed for Edge Impulse, a machine learning development platform geared towards edge devices [16]. These solutions specifically target ultra-low power sensor solutions for wearables and the Industrial Internet of Things, demonstrating the relentless commitment to pushing the boundaries of technological innovation. In a rapidly evolving landscape, neuromorphic computing is at the forefront of shaping the future of robotics and advanced technological solutions.

## 3. Neuromorphic Photonic Integrated Circuits

With the recent emergence of Photonic Integrated Circuit (PIC) technology platforms, the timing is perfect for developing scalable, fully reconfigurable systems capable of executing vastly more complex operations than ever before [17]. While numerous fields, such as microwave photonics and physical layer security, stand to benefit significantly from this rapid increase in complexity, the community has yet to establish a universal processing standard for programming intricate multistage operations within the photonic domain. Neuromorphic photonics is an exciting and emerging field at the intersection of neuroscience and photonics. This groundbreaking discipline harnesses the efficiency of NNs and the lightning-fast capabilities of photonics to create processing systems that can outperform microelectronics by orders of magnitude. Thanks to their partial analog nature, neuromorphic circuits can leverage optical signals’ vast bandwidth and energy efficiency. Additionally, they set the stage for a comprehensive processing standard for reconfigurable circuits capable of theoretically executing any task that an artificial NN can compute. Integrating these systems with low-power microelectronic control promises processing efficiencies that surpass current digital standards by a considerable margin. In essence, the emergence of PIC technology, coupled with the advent of neuromorphic photonics, heralds a new era of computing where the potential for innovation and efficiency is boundless.

To transcend the constraints imposed by traditional microelectronic computing, it is imperative to incorporate unconventional techniques that leverage new processing methodologies. PICs offer a promising avenue to address these limitations, and several factors underscore their suitability. Firstly, photonic interconnects present a direct solution to the data transport quandary: a substantial portion of energy consumption on modern microelectronic chips is attributed to metal wires’ constant charging and discharging. This energy overhead can be circumvented by using on-chip photonic links, especially as optical devices advance in efficiency [18]. Secondly, photonic systems can harness optical multiplexing and high-speed signals to achieve an impressive bandwidth density. This translates into a remarkable computational density (operations per second per square millimeter, ops/s/mm^2^) for closely spaced waveguides or filters that perform densely packed operations [19].

**Figure 2:**
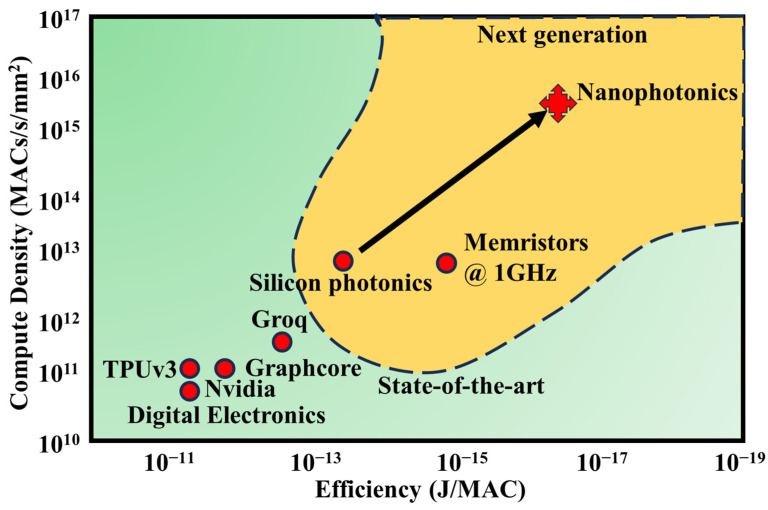
A comparison between specialized deep-learning digital electronic architectures and silicon photonic and nanophotonic platforms. In this context, photonic systems can support high on-chip bandwidth densities while maintaining low energy consumption during data transmission and computational tasks. The metrics for electronic architectures have been sourced from various references [21,22,23,24]. The metrics for silicon photonic platforms are estimated based on a contemporary silicon photonic setup operating at 20 GHz, comprising 100 channels with tightly packed micro rings. Meanwhile, the nanophotonic metrics are derived from the assumption of closely packed athermal microdisks [25], each occupying an area of approximately 20 µm, running at 100 GHz and operating close to the shot noise limit. Inspired by [17].

Furthermore, implementing linear operations like Multiply-Accumulate (MACs) in the photonic realm inherently consumes minimal energy, yielding a highly advantageous, sublinear scaling of energy consumption concerning the number of operations conducted [20]. The combination of these three properties can deliver substantial enhancements in performance, encompassing energy efficiency and computational density, as illustrated in Figure 2.

Neuromorphic photonic systems have demonstrated processing speeds 6–8 orders of magnitude higher than their electronic counterparts [26]. Silicon photonics, an optoelectronic integration technology compatible with well-established microelectronics, harmonizes the ultra-large-scale logic and precision manufacturing attributes of CMOS technology with the high-speed and low-power consumption benefits of photonic technology, effectively reconciling the conflict between technological advancement and cost constraints. In recent years, on-chip NNs based on silicon photonic technology have made significant strides [27]. In 2017, Shen et al. showcased an on-chip NN employing a silicon-based Mach–Zehnder interferometer structure capable of recognizing fundamental vowels [20]. In this architecture, an external subsystem configures the matrix element values for vector-matrix multiplication using Mach–Zehnder interferometer (MZI) structures. To modify these values during optimization, signals must be relayed from the NN to the control system. Tait et al. introduced on-chip variable weight synapses based on silicon electro-optical modulators in 2016 [28], as well as on-chip neurons relying on silicon electro-optical modulators in conjunction with off-chip multi-wavelength lasers, wavelength division multiplexers/demultiplexers, and on-chip photodetectors in 2019 [29]. This innovative structure facilitates weight adjustments by modulating the silicon microring with electrical signals and regulates the silicon microring modulator to achieve neuron functionality through electrical signals derived from on-chip detector optoelectrical conversion.

Neuromorphic PICs on silicon platforms have witnessed remarkable advancements in recent times [26,30,31,32]. These photonic NNs (PNNs), even in their early stages with a limited number of neurons, have showcased their prowess in high-bandwidth, low-latency machine-learning signal processing applications. The next frontier in this domain involves the quest for large-scale PNNs endowed with flexibility and scalability, positioning them to tackle data-intensive machine learning (ML) applications with high-speed requirements. In [33], architectural foundations are proposed, focusing on microring resonator (MRR)-based photonic neurons, both non-spiking and spiking, and the orchestration of PNNs through a broadcast-and-weight approach. A novel expansion of NN topologies by cascading photonic broadcast loops is discussed, culminating in a scalable NN structure with consistent wavelengths. Moreover, incorporating wavelength-selective switches (WSS) within these broadcasting loops is proposed, delivering the concept of a wavelength-switched photonic NN (WS-PNN). This innovative architecture opens new doors for integrating off-chip WSS switches, enabling the interconnection of photonic neurons in versatile combinations, delivering unmatched scalability for PNNs, and accommodating an array of feedforward and recurrent NN topologies.

### 3.1. Deep DNNs

Deep neural networks (DNNs) have gained prominence due to advancements in processing power and the ubiquity of data. Faster and more affordable computing resources have facilitated rapid convergence, making deep learning (DL) more accessible. The widespread availability of data, along with improved algorithms, enhances the value of these networks, especially in applications like chatbots for businesses [34,35]. These networks, however, demand substantial computational power and extensive data sets. They excel in scenarios where ample data is available and where it is feasible to categorize or rank preferred outcomes [4].

**Figure 3:**
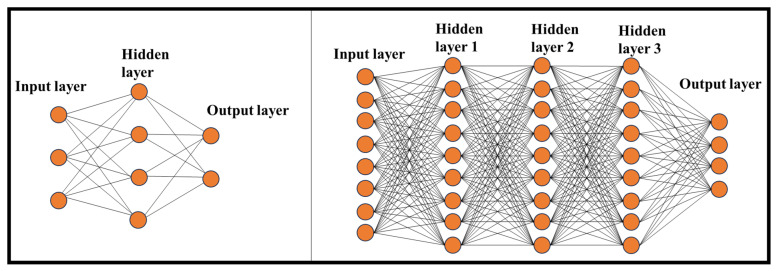
Traditional NN (**left**) versus DNN (**right**).

DNN represents a sophisticated machine learning (ML) technique that empowers computers, through training, to accomplish tasks that would be exceedingly challenging with traditional programming methods [36]. The inspiration for NN algorithms is drawn from the human brain and its intricate functions. Like the human mind, DNNs are designed not to rely solely on predetermined rules but to predict solutions and draw conclusions based on previous iterations and experiences. A NN consists of multiple layers of interconnected nodes that receive input from previous layers and generate an output, ultimately reaching a final result. NNs can encompass various hidden layers, and the complexity increases with adding more layers. Here are distinct neural network architectures (Figure 3):

(A) Traditional NNs: Typically composed of 2 or 3 hidden layers.
(B) DL Networks: These can contain up to 150 hidden layers, making them significantly more complex.

A DNN is considerably more intricate than a “simple” NN. A standard NN operates akin to a chess game, adhering to predefined algorithms. It offers different tactics based on inputs from the programmer, such as how chess pieces move, the size of the chessboard, and strategies for various situations. However, a NN transcends this input-bound behavior and can learn from past experiences, evolving into a DNN. For instance, on the same computer, you can train an NN, play games against other individuals, and enable it to learn as it engages in these matches. As it learns from various players, defeating a DNN, even for chess masters, might become exceedingly challenging or even insurmountable. DNNs can recognize voice commands, identify voices, recognize sounds and graphics, and accomplish a wide array of tasks beyond the capacity of traditional NNs. They leverage “big data” along with sophisticated algorithms to tackle complex problems, often requiring minimal to no human intervention.

Understanding the process of a DNN is best illustrated through a practical example. Imagine you have an extensive collection of hundreds of thousands of images, some of which feature dogs, and you aim to create a computer program to identify dogs in these pictures. At this point, you face a crucial decision. You can either write a program explicitly designed to identify dogs or opt for a more intelligent approach—a program that “learns” how to recognize dogs. Initially, you might choose the former option, but this turns out to be a less-than-ideal choice. Conventional programming techniques require a laborious and intricate process, and the outcomes often lack the desired accuracy. To explicitly identify dog pictures, you must create a software program filled with conditional “if” and “then” statements. This program would elevate the probability of a dog’s presence whenever it detects a dog-like attribute, such as fur, floppy ears, or a tail.

Convolutional neural networks (CNNs) represent a subset of AI explicitly designed to handle and learn from vast datasets. These networks are aptly named due to their distinctive architecture and purpose. CNNs excel in image recognition and perform not only generative but also descriptive tasks. Generative tasks encompass various activities such as auto-cropping, caption generation, video processing, mimeographing, and image overlays. A vital component of a CNN is the convolutional layer, where each neuron processes information from a small portion of the visual field, with their inputs forming a checksum-like pattern to create feature maps.

Artificial neural networks (ANNs) are interconnected perceptrons organized into various layers. ANNs are often called Feedforward Neural Networks, as they process inputs linearly, forwarding the results through the network layers. These networks are known as universal function approximators, capable of learning any function, and their versatility is attributed, in part, to activation functions. These functions introduce nonlinearity into the network, enabling it to learn intricate relationships between inputs and outputs and promoting cooperative learning among network parts. It is important to note that the logic behind neural networks is often incomprehensible to humans. Deep learning models operate as black boxes, with hidden layers of nodes creating complex, interconnected logic. Some attempts have been made to visualize the logic behind NNs for image recognition, but this is not always possible, especially for demanding tasks.

### 3.2. NNs with Complex Arithmetic Calculations

While computers excel at performing complex calculations, the realm of solving mathematical problems continues to present a significant challenge for artificial intelligence [37]. This challenge can be viewed from two distinct angles. On the one hand, grounding structured mathematical knowledge into a framework of intrinsic meaning has persisted as a longstanding issue in symbolic AI [38]. On the other hand, NNs have traditionally struggled to acquire mathematical proficiency, as their nature primarily hinges on statistical pattern recognition abilities rather than the explicit application of syntactic rules [39]. The process of mathematical reasoning poses well-documented hurdles for connectionist models. Mathematical formulas employ symbols that often appear as arbitrary tokens, necessitating manipulation under well-defined rules that involve compositionality and systematicity. Furthermore, extracting mathematical knowledge from examples should extend beyond the observed data distribution, facilitating the ability to extrapolate by discovering fundamental ‘first principles’.

Notwithstanding these formidable challenges, recent breakthroughs in DL have sparked a renewed enthusiasm for the notion that NNs may attain advanced reasoning capabilities, consequently displaying symbolic behavior [40]. Although deep networks have historically grappled with fundamental concepts such as the understanding of ‘integer numbers’ [41], the last few years have witnessed the emergence of several models that showcase remarkable proficiency in tackling intricate mathematical tasks.

For instance, sequence-to-sequence architectures have demonstrated their ability to learn the intricacies of function integration and the resolution of ordinary differential equations, occasionally outperforming even widely used mathematical software packages in terms of accuracy [42]. DL models have further made notable inroads in the realm of automated theorem proving [43] and have actively supported expert mathematicians in the formulation of conjectures and the establishment of pioneering results in the realm of pure mathematics [44].

In remarkable developments from last year, deep reinforcement learning uncovered a more efficient algorithm for performing matrix multiplication [45], while fine-tuning a pre-trained language model on computer code enabled the resolution of university-level mathematical problems at a level comparable to human expertise [46]. These achievements herald a promising new era where neural networks may bridge the gap between mathematical reasoning and machine learning, potentially unlocking new frontiers in artificial intelligence.

These outstanding accomplishments owe much to the advent of meticulously curated, expansive datasets encompassing mathematical problems and their corresponding solutions. Furthermore, they owe their success to inventing novel, sometimes ad hoc, architectures tailored to more effectively process numerical symbols and mathematical notations. In addition, strides in many tasks have been propelled by creating large-scale language models, which exhibit astonishing innate numerical capabilities ‘out of the box’, that can be further honed through fine-tuning and strategic prompting techniques.

However, it is imperative to emphasize that these achievements do not necessarily equate to a full grasp of the semantics underlying numbers and basic arithmetic by these models. Their performance on relatively straightforward numerical tasks often reveals fragility, signaling a need to enhance their foundational mathematical skills to establish a more dependable foundation for mathematical capabilities. This notion finds support in a wealth of literature on child development and education, which underscores the significance of fundamental numeracy skills such as counting, quantity comparison, comprehension of number order, and mastery of the base-ten positional numeral system as robust predictors of later mathematical achievement [47].

The quest for solutions to matrix eigenvalues has perpetually been a focal point of contemporary numerical analysis, with profound implications for the practical application of engineering technology and scientific research. While extant algorithms for matrix eigenvalue computation have made considerable progress in computational accuracy and efficiency, they have struggled to find a foothold of photonic platforms. Enter the PNN, a remarkable fusion of potent problem-solving capabilities and the inherent advantages of photonic computing, characterized by its astonishing speed and minimal energy consumption. In [48], an innovative approach introduces an eigenvalue solver tailored for real-value symmetric matrices, leveraging reconfigurable PNNs. This strategy demonstrates the practicality of solving eigenvalues for n × n real-value symmetric matrices using locally connected networks. In a groundbreaking series of experiments, the capacity to solve eigenvalues for 2 × 2, 3 × 3, and 4 × 4 real-value symmetric matrices through the deployment of graphene/Si thermo-optical modulated reconfigurable photonic neural networks featuring a saturated absorption nonlinear activation layer was showcased. Theoretical predictions indicate a remarkable test set accuracy of 93.6% for 2 × 2 matrices, with experimental results achieving a measured accuracy of 78.8%, aligning with standardized metrics for easy comparison. This work not only charts a course for on-chip integrated photonic solutions to eigenvalue computation for real-value symmetric matrices but also forms the bedrock for a new era of intelligent on-chip integrated all-optical computing. This breakthrough promises to transform the landscape of computational methodologies, ushering in a future where photonic platforms play a pivotal role in numerical problem-solving across various domains [48].

**Figure 4:**
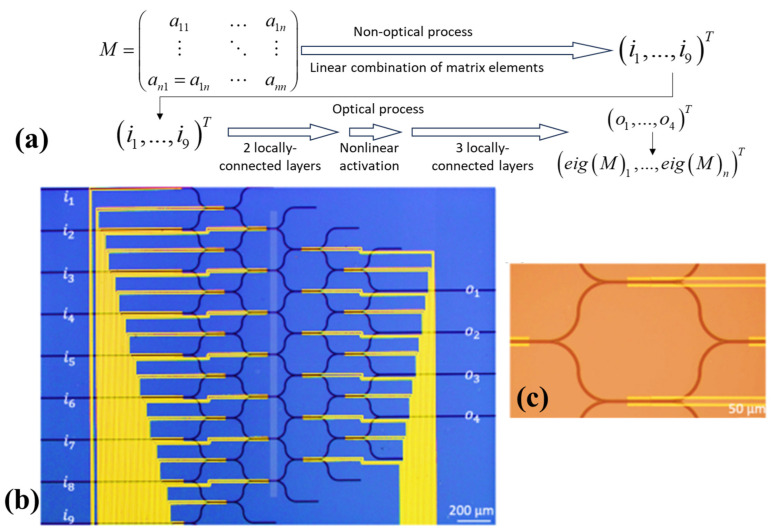
Conceptual framework of the newly proposed photonic neural network. The essential steps involved in achieving the desired task (**a**), an optical micrograph showcasing the distinctive structure of the proposed network, featuring nine input ports (*i*_1_–*i*_9_) and four output ports (*o*_1_–*o*_4_) [48] (**b**), an optical micrograph that zooms in on a single cell within the network, housing two phase shifters and a merging structure [48] (**c**).

The objective of the proposed PNN is to address the challenge of computing eigenvalues for symmetric matrices. This problem frequently arises in the context of various physical scenarios (as shown in Figure 4a). The initial focus centers on solving the eigenvalue problem for 2 × 2 symmetric matrices characterized by non-negative real-value elements and eigenvalues. Furthermore, the matrix elements were confined within the range of 0 to 10. This limitation does not constrain the network’s performance, as any other matrices can be derived through linear scaling from a matrix within this constrained domain. Crucially, this network is adaptable and designed to handle the eigenvalue problem for n × n matrices under similar conditions. This versatility allows it to be employed in diverse scenarios, offering a powerful tool for eigenvalue computation in various applications.

The structure of the PNN is characterized by an architectural design that includes a single linear fully connected layer, complemented by a sophisticated five-layer locally connected arrangement. This network boasts nine input and four output ports, as Figure 4b depicts. The five-layer structure is a critical component of the described architecture, characterized by an intricate arrangement of neurons. In the first layer, eight neurons are featured, each sharing a phase shifter with its neighboring unit (as illustrated in Figure 4c).

The next layer comprises seven neurons, with each successive layer reducing the count by one, resulting in 35 tunable weights. Additionally, the authors of this work introduced two extra weights for training. The first weight pertains to the input light’s intensity, denoting the intensity ratio. This factor is crucial as the nonlinear activation function behaves differently under varying intensities. The second weight governs the output ratio, linearly adjusting the relationship between output intensity and the corresponding eigenvalue, effectively establishing the output ratio. This adjustment is essential because, unlike electronic neural networks, optical layers cannot manipulate light intensity freely and directly. Consequently, the absolute value of the output signal may not align with the scale provided in the dataset.

**Figure 5:**
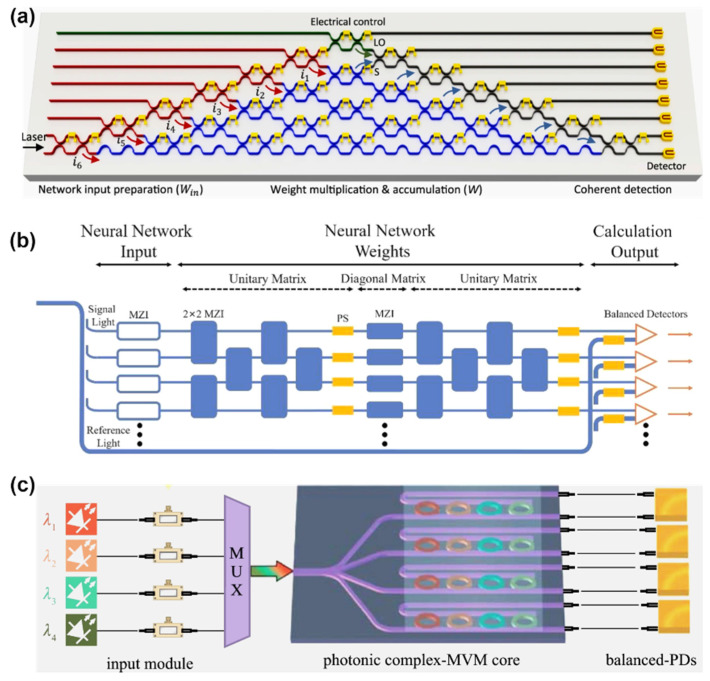
The developed architectures of complex-valued optical neural architectures using: (**a**,**b**) MZIs, the circuits themselves realize a multiport interferometer with phase shifters (PSs) inserts used for phase tuning [49,50]; (**c**) MRRs for matrix-vector multiplication (MVM) applications using WDM [51].

Photonic circuits have also found their applicability in complex-valued neural networks [49,50,51]. The articles [49,50] presented neural network architectures that use complex arithmetic computations and a MZI to encode information in both phase and amplitude (Figure 5a,b). This approach allows complex arithmetic to be performed using the properties of interference. The resulting complex-valued ONCs (optical neural chips) perform better on several tasks than their counterparts in single-neuron and deployed network implementations. A single complex-valued neuron can solve some nonlinear problems that a real-valued analog cannot compute. There are many comparative analyses, tests, and trainings of the NN on various datasets. The data obtained suggest that this architecture uses double the number of trained free parameters, and can classify nonlinear patterns with simple architectures (fewer layers). Research results have shown that these architectures significantly improve the speed and accuracy of computation compared to traditional real-valued circuits.

The application of MRR arrays in complex-valued neural networks is also possible, as demonstrated in [51]. To realize the transition from real values to complex-valued data, an approach with a pre-decomposition of the input matrix (the values are supplied to beams with different wavelengths employing optical intensity modulators) and the transmission matrix (controlled by selection of values utilizing heaters on the resonator rings) (Figure 5c) is used in this work. A balanced photodetector registers the result of the multiplication of the two matrices. This approach allowed the realization of other mathematical transformations, including discrete Fourier transform (DFT) and convolutional image processing. The results of the experiments in both signal and image processing unequivocally show that the newly proposed system can expand matrix computation to include real numbers, full complex numbers, higher processing dimensions, and convolution. Consequently, the processor can function as a versatile matrix arithmetic processor capable of handling intricate tasks in different scenarios. The authors note that improved system performance can be obtained by adding parallel computation with WDM and increasing the degree of integration of the circuit components.

### 3.3. Spike NNs

Over the past decade, ANNs have made remarkable strides, progressing from the initial multi-layer perceptron (MLP) of the first generation to the cutting-edge techniques of the second-generation DNNs [52,53]. This advancement has been significantly fueled by abundant annotated data and the widespread availability of high-performance computing devices, including versatile Graphics Processing Units (GPUs). However, even with these achievements, ANNs still fall short of matching biological neural networks’ (BNN) energy efficiency and their online learning capabilities. Many endeavors have been undertaken to diminish the power consumption of conventional deep-learning models. These efforts aim to uncover more streamlined networks that deliver similar performance with reduced complexity and fewer parameters than their original counterparts. Several techniques have been developed for this purpose, including quantization [54], pruning [55], and knowledge distillation [56]. Quantization involves converting the network’s weights and inputs into integer types, thereby lightening the overall computational load. Pruning entails the iterative removal of connections within a network during or after training to compress the network without compromising performance. Knowledge distillation transfers the intricate knowledge acquired by a high-complexity network, the teacher, to a lightweight network known as the student.

While ANNs and DNNs have traditionally been inspired by the brain, they fundamentally differ in structure, neural computations, and learning rules compared to BNNs. This realization has led to the emergence of spiking neural networks (SNNs), often regarded as the third generation of NNs, offering the potential to surmount the limitations of ANNs. The utilization of SNNs on neuromorphic hardware like TrueNorth [57], Loihi [58], SpiNNaker [59], NeuroGrid [60], and others presents a promising solution to the energy consumption predicament. In SNNs, similar to BNNs, neurons communicate via discrete electrical signals known as spikes and operate continuously in time. Due to their functional resemblance to BNNs, SNNs can exploit the sparsity inherent in biological systems and are highly amenable to temporal coding [61]. While SNNs may still trail behind DNNs regarding overall performance, this gap is narrowing for specific tasks. Notably, SNNs typically demand considerably less energy for their operations. Nevertheless, training SNNs remains challenging due to the intricate dynamics of neurons and the non-differentiable nature of spike operations.

### 3.4. Convolutional Neural Networks (CNNs)

**Figure 6:**
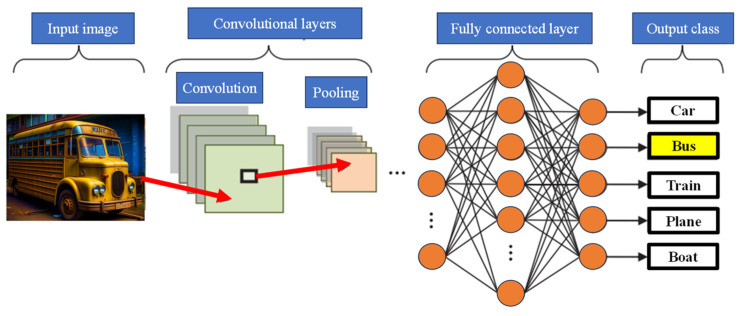
CNN image classification pipeline.

CNNs are inherently feedforward networks, exhibiting unidirectional information flow, transmitting data exclusively from inputs to outputs. As ANNs draw inspiration from biological systems, CNNs share a similar motivation. Their architecture is heavily influenced by the brain’s visual cortex structure, characterized by layers of simple and complex cells [62,63]. CNN architectures offer a range of variations yet generally comprise convolutional and pooling (subsampling) layers organized into distinct modules. These modules are subsequently followed by one or more fully connected layers, resembling a conventional feedforward NN. Often, these modules are stacked to create deep models. Figure 6 illustrates typical CNN architecture for a simplified image classification task, where an image is initially fed into the network and undergoes several convolution and pooling stages. The representations obtained from these operations are then channeled into one or more fully connected layers. Finally, the last fully connected layer provides the output as a class label. While this architecture remains the most prevalent in the literature, various changes have been proposed in recent years to enhance image classification accuracy or economize on computation costs.

CNNs represent a revolutionary paradigm shift in image recognition, enabling the detection and interpretation of intricate patterns within visual data [64]. Their effectiveness is unrivaled, positioning them as the preeminent architecture for image classification, retrieval, and detection tasks, delivering results characterized by exceptional accuracy. The versatility of CNNs extends to real-world scenarios, where they consistently yield high-quality results. They excel in localizing and identifying objects, be it a person, a car, a bird, or any other entity within an image. This adaptability has made CNNs the default choice for predictive image input tasks. A fundamental attribute of CNNs is their capacity to attain ‘spatial invariance’. This signifies their ability to autonomously learn and extract image features from any location within the image, obviating the need for manual feature extraction. CNNs draw these features directly from the image or data, underscoring their potency within the realm of DL and their remarkable precision. As elucidated in [65], the purpose of pooling layers is to reduce the spatial resolution of feature maps, thereby achieving spatial invariance to input distortions and translations. Pooling layers streamline image processing and enhance computational efficiency by reducing the number of required parameters, resulting in expedited data processing. This reduction in memory demands and computational costs bolsters the appeal of CNNs. While CNNs have prominently left their mark on image analysis, their scope extends well beyond this domain. They can be applied to diverse data analysis and classification challenges. This adaptability spans various sectors, yielding precise outcomes in face recognition, video classification, street and traffic sign recognition, galaxy classification, and the interpretation and diagnosis of medical images, among others [66,67,68].

### 3.5. Methods for Implementing the Activation Functions in Optical Neural Networks

AI has become instrumental across diverse applications. Nevertheless, AI systems traditionally demand substantial computational resources and memory. The diminishing returns of Moore’s law have signaled a shift away from conventional architectures for AI algorithms, as referenced in [69]. Furthermore, the pressing need for power-efficient implementations of ANNs has surfaced, particularly in scenarios like image recognition, where processing a single image may entail billions of operations [70]. There is an active exploration into replacing or supplementing traditional integrated electronic circuits with photonic circuits. A pivotal facet of silicon photonics is WDM, which empowers the simultaneous transmission of multiple signals over a shared medium without interference. In Optical Neural Networks (ONNs), WDM facilitates parallel processing of multiple data streams simultaneously. ONNs promise to surpass their electronic counterparts in terms of both speed and energy efficiency. For instance, common operations like matrix multiplications are resource-intensive on conventional computers, but they can be executed at ultra-high speeds using specialized configurations of photonic networks [71]. All-optical ANNs, devoid of optoelectronics or electro-optical conversion other than the interface, enable matrix multiplications to occur at the speed of light as optical signals propagate through waveguides. Silicon photonics further allows the integration of photonic and electronic devices on the same platform [72].

In this context, two prominent optical modulators, Mach–Zehnder interferometers (MZIs) and microring resonators (MRRs), are commonly employed [73,74]. MZIs, although bulkier, exhibit resilience to process and temperature variations due to their signal processing method, which involves signal delay within one of the two branches. On the other hand, MRRs are more compact and rely on slight detuning of the resonant wavelength from the input signal to perform dot products. This approach enables WDM but introduces challenges related to the accurate calibration of the resonant rings, as their resonance can drift with temperature variations, leading to increased complexity and power overhead.

Replicating an ANN with an Optical Neural Network (ONN) presents a significant challenge, primarily revolving around the comprehensive optical implementation of every core module in a conventional ANN. While optical matrix multiplication has been successfully realized [75], the activation function (AF), a pivotal element in ANNs, remains a complex issue. The matrix multiplication stage corresponds to the linear transformation data undergo in an ANN. However, to achieve optimal results, a non-linear transformation is equally essential, typically performed by the AF. Existing contributions in this domain have taken different approaches. Some ONN implementations incorporate the AF through computer-based or partially electrical components. In contrast, others strive for full optical integration by utilizing optical non-linearities at either a material or device level. In the former approach, the optical circuit’s information is converted into electrical format for AF processing on a computer, and then the output is reconverted into the optical circuit. However, this method limits the network’s speed due to electronic circuit constraints, introducing noise that degrades accuracy. Moreover, this dual conversion process introduces considerable latency and higher power consumption, ultimately undermining the advantages of optical implementation.

Despite the introduced network delays and significant increases in power consumption and chip size, the O-E-O conversion remains the most common way to implement the activation function on a photonic chip. Since achieving nonlinearity of the characteristic only on photonic elements is a challenging task, many researchers are developing various combinations of photonic elements that can influence the characteristic for the necessary adjustment by electronic components. Also, solutions include using hybrid structures (Ge/Si hybrid structure in a micro-ring resonator) [76], structures using the free-carrier dispersion effect (scheme with a Mach–Zehnder interferometer loaded with MCR, heating elements, and a Mach–Zehnder coupler) [76,77,78] and another popular direction—phase change material (PCM) coatings [79,80]. The given examples can realize not one but several variants of activation functions: radial basis, sigmoid, softplus, Relu, and ELU. This increases the flexibility of these structures because, depending on the task solved by the neural network, different characteristics and threshold values of activation functions may be required.

Consequently, despite the promising results achieved by works that implement the AF electrically [81,82], it is believed that an optical AF is imperative to unlock the full potential of ONNs. Such an approach can mitigate the bottlenecks associated with electronic conversions and offer the speed, precision, and efficiency required to fully harness the capabilities of ONNs.

Nevertheless, the implementation of AFs in optical networks can diverge due to the inherent nature of optical computing. Several standard optical activation functions are employed in these systems. One approach involves Nonlinear Optical Activation; whereby optical components are deliberately engineered to demonstrate nonlinear behavior. Notable examples include the Kerr effect and cross-phase modulation, both of which enable the creation of nonlinear optical activations by nonlinearly modulating the intensity of the light field [83]. Optical bistability is another avenue, employing optical bistable devices as activation functions. These devices exhibit two stable states and can be manipulated by adjusting input power or other optical parameters, thus serving as activation elements [84]. Optical switches come to the fore in the realm of all-optical Switches [85]. These switches can be deployed to execute binary-like activation functions by altering the optical signal’s path or state based on input intensity, rendering them well-suited for binary activations within optical neural networks. MZIs represent yet another option, capable of generating optical interference patterns sensitive to input intensity [86]. Through controlled phase shifts in the interferometer, they can be harnessed to perform activation functions. Nonlinear crystals offer a different route, enabling the creation of optical parametric amplifiers and oscillators and introducing nonlinear activation functions within photonic neural networks [87]. Lastly, resonators like ring resonators can be incorporated as activation functions, capitalizing on their resonance properties and input power levels [88].

The choice of an optical activation function in photonic neural networks hinges on the specific architectural design, hardware components, and the intended network characteristics. These optical activation functions are engineered to carry out nonlinear operations on optical signals, mirroring the behavior of digital activation functions found in conventional neural networks. Optical neural networks remain an active arena of research, continually producing novel techniques for implementing optical activation functions.

### 3.6. Programmable PNNs

The rapid and explosive growth of AI and Deep Learning (DL), coupled with the maturation of photonic integration, has opened a new realm of possibilities for optics in computational tasks [89,90]. Applying photons and advanced optical technologies in Neural Network (NN) hardware holds immense promise. It is projected to substantially increase Multiply-Accumulate (MAC) operations per second compared to traditional NN electronic platforms. Computational energy efficiency is estimated to plummet below the femtojoule (fJ) per MAC mark, while the area efficiency is anticipated to soar beyond millions of MAC operations per square millimeter [91,92]. This paradigm shift in NN hardware seeks to leverage the high data transmission rates enabled by integrated photonic technologies while also harnessing the compact size and low power consumption capabilities inherent to chip-scale designs. Up until now, the predominant focus in photonic devices designed for weight calculations has centered around elements that can be slowly reconfigured, such as Thermo-Optic (T/O) phase shifters [50] and Phase-Change Material (PCM)-based non-volatile memory structures [89]. This emphasis on slow reconfiguration implies that inference applications currently take precedence in neuromorphic photonics [26].

Extending reconfiguration capabilities to Photonic (P)-NN implementations demands a platform that can accommodate various functional layouts within the same neural hardware. Over the past few years, the realm of photonics has made significant strides in programmability [93], and programmable PICs [94] have emerged as a pivotal resource for fostering cost-effective, versatile, and multifunctional photonic platforms, akin to the concept of electronic Field-Programmable Gate Arrays (FPGAs) [93]. Furthermore, it has been demonstrated that merely incorporating slowly reconfigurable Mach–Zehnder Interferometric (MZI) switches within a suitable architectural framework can provide a plethora of circuit connectivity and functional possibilities [93]. Nonetheless, the unique characteristics of NN architectures necessitate the exploration of alternative functionalities yet to be covered by programmable photonic implementations. While contemporary photonic weighting technology can indeed facilitate weight value reconfiguration [20], there is a growing shift towards considering programmable activation functions [95]. Nevertheless, it is essential to note that existing neuromorphic photonic architectures lack reconfiguration mechanisms for their linear neuron stages. Photonic Neural Networks (PNNs) have mainly advanced within two primary architectural categories for implementing linear neural layers. The first category involves incoherent or Wavelength-Division-Multiplexed (WDM) layouts, where each axon within the same neuron is assigned a distinct wavelength [96]. The second category centers on coherent interferometric schemes, in which a single wavelength is utilized throughout the entire neuron, harnessing interference between coherent electrical fields to perform weighted sum operations.

An innovative architecture is proposed in [97] that seamlessly integrates WDM and coherent photonics to empower Programmable Photonic Neural Networks (PPNNs) with four distinct operational modes for linear neural layers. Building upon their previously proposed dual-IQ coherent linear neuron architecture [98], which recently demonstrated remarkable computational performance as a PIC with groundbreaking compute rates per axon [99], their next step is advancing single neuron architecture. This approach involves harnessing multiple wavelength channels and corresponding WDM De/Multiplexing (DE/MUX) structures to create multi-element and single-element fan-in (input) and weight stages for each axon. Programmability is achieved by integrating Mach–Zehnder Interferometer (MZI) switches, which can dynamically configure the connections between fan-in and weighting stages, offering the flexibility to define neural layer topologies through software.

A comprehensive mathematical framework for this programmable neuromorphic architecture was established and delved into a thorough analysis of potential performance limitations associated with using multiple wavelengths within the same interferometric arrangement. These findings led to a straightforward mechanism to mitigate wavelength-dependent behaviors in modulators and phase shifters at the fan-in and weighting stages. As a result, this programmable layout consistently delivers exceptional performance across all four distinct operational modes, ensuring that supported neurons always maintain a relative error rate lower than a specified threshold, provided that inter-channel crosstalk remains within the typical range of values below a certain threshold.

**Figure 7:**
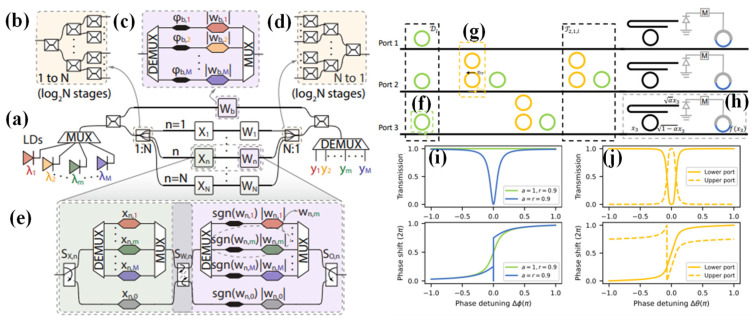
(**a**) An illustration of the PPNN. It consists of several components, including M laser diodes (LDs), a MUX, a 3dB X-splitter, a bias branch denoted as W_b_, and a reconfigurable Optical Linear Algebra Unit (OLAU) [97]. The OLAU comprises a 1-to-N splitting stage, input (X_n_) and weight (W_n_) modulator banks, and an N-to-1 combiner stage. The output from the combiner stage interferes with the bias signal within a 3dB X-coupler and is then sent to a DEMUX. A closer examination reveals details of (**b**) 1-to-N splitting and (**d**) its N-to-1 coupling stage [97], (**c**) view of the bias branch, which includes wavelength-selective weights and phase modulators [97], (**e**) a closer look at an axon of the OLAU, which consists of switches for signal routing and modulators for inputs (x_n_,m) and weights (w_n_,m) [97], layout of a Single Layer Coherent Optical Neural Network [106] (**f**) a tunable all-pass single RR functions as a phase tuning component, (**g**) tunable serially-coupled double RRs are employed as signal mixing components between the ports, (**h**) the nonlinear activation unit transforms input signal 𝑥𝑛 into 𝑓(𝑥𝑛), where 𝑓 represents a nonlinear function (with 𝑛 = 3 in this example). The black ring within the nonlinear activation unit acts as a directional coupler, directing a portion of the optical energy (𝛼) for electrical signal processing. The diode is a photodetector, and the blue ring modulates the signal. An electronic circuit (M) processes the electronic output from the photodetector to generate a modulation signal for the right ring [106] (**i**) displays the transmission and phase responses of a bus waveguide side-coupled with a ring, showcasing variations as a function of phase detuning, Δ*ϕ*. Over-coupling, indicated in green, is employed for phase-tuning components. At the same time, critical coupling, highlighted in blue, is crucial for achieving a larger amplitude tuning range in the nonlinear activation ring [106], (**j**) provides an example transmission and phase response of the coupled double ring, used as a signal mixing component [106].

Figure 7a [97] depicts the fundamental structure of the neural layer. Instead of a single Continuous Wave (CW) input optical signal, M multiplexed CW signals are each centered at λ_m_ and dedicated to an independent virtual neuron. The input and weight modulators have been replaced by more intricate modulator banks, as illustrated in Figure 7c,e. Software-controlled switches enclose these modulator banks. The multichannel input signal is divided into two portions in the initial stage. One portion is directed to the bias branch, while the remaining part enters the Optical Linear Algebraic Unit (OLAU). Within the OLAU, the signal undergoes further splitting, with equal power distribution achieved by a 1-to-N splitter, an example of which is provided in Figure 7b. Subsequently, after being appropriately modulated by inputs (x_n,m_) and weighted by (w_n,m_), the signal is routed to the N-to-1 combiner, as depicted in Figure 7d [97]. At this juncture, the output signal interferes with the bias signal within a 3 dB X-coupler and is then directed to the DEMUX to generate the outputs (y_m_). In the final step, each channel (m) undergoes algebraic addition of the weighted inputs with a designated bias. This results in a total of M independent N-fan-in neurons.

Many cutting-edge programmable photonic circuits leverage the remarkable capabilities of Mach–Zehnder interferometers (MZIs). MZIs offer precise control over power splitting ratios and relative phase shifts between input and output ports, achieved by adjusting the phase-shifting control elements using either thermo-optic or electro-optic effects. Through the strategic combination of multiple directional couplers and phase shifters within specific mesh configurations [100,101], MZI-based architectures can perform a diverse array of linear transformations across various ports. When complemented by optic-electro-optic nonlinearity [20] or optical-modulator-based reprogrammable nonlinearity [102], MZI-based architectures have proven their mettle in tackling intricate machine learning tasks, boasting superior processing speeds. Nevertheless, in the pursuit of significant phase tuning ranges, MZIs demand relatively high driving voltages [103], and the devices can extend up to around 100 μm in length. In large-scale on-chip integrated circuits designed for complex applications, two vital factors emerge as primary concerns: the device’s footprint and power consumption. A natural and promising avenue is the adoption of resonant structures that enhance light-matter interactions, thereby reducing device footprint, driving voltages, and overall power consumption [103].

Among these, MRRs have garnered attention for their ability to program real-valued weights through a ‘broadcast-and-weight’ protocol [104], resembling a continuous-time recurrent neural network [30]. A notable advancement involves programming weights at the interconnected waveguides between two MRRs using phase-change materials. This innovation has led to the development of a photonic tensor core, serving as a robust dot-product engine [105]. It is worth mentioning that most prior proposals employing MRRs primarily relied on wavelength-division multiplexing for input signals, and incoherently aggregated signals at the photodetectors. The potential of coherence networks, which harness the wave nature of electromagnetic fields, holds promise for novel advancements in the design of optical neural networks [50].

A groundbreaking coherent optical neural network architecture built upon MRRs is proposed in [106]. This innovative approach offers notable advantages regarding device footprint and energy efficiency compared to conventional optical neural networks based on Mach–Zehnder interferometer (MZI) architectures. This architecture’s linear matrix multiplication layer is fashioned by linking multiple linear units, each comprising a serially coupled double-RR [107] for harmonizing signals from different ports and a single-RR for precise phase adjustments. Incorporating element-wise activation at each port, this nonlinear unit is crafted using microring modulators and electrical signal processing, granting the flexibility to program diverse nonlinear activation functions. Notably, the linear and nonlinear components presented in this work maintain the coherency of input signals, thus constituting a complex-valued neural network [50]. Moreover, the inherent flexibility of this design enables the direct cascading of each layer on the same chip without the need for intermediate digital-to-analogue conversions. This reduces latency and minimizes energy waste associated with signal conversions. The input-output relationship in the designed architecture was illustrated through a transfer function, and automatic differentiation was employed [27,28] to train the tunable parameters directly. The design and training algorithms are not confined to the ring-based MRR design and can be adapted to various tunable systems. The network’s proficiency in information processing tasks was showcased to provide a concrete example of its capabilities, such as functioning as an Exclusive OR (XOR) gate and conducting handwritten digit recognition using the MNIST dataset [108].

In [106], ring-based programmable coherent optical neural network configuration is presented, as illustrated in Figure 7. Figure 7f,g are dedicated to the fundamental elements responsible for executing the linear transformation, described by the matrix 𝑊_𝑙_. In contrast, Figure 7h represents the component running nonlinear activation functions. These components are constructed using waveguides that are intricately coupled to RRs. It is noteworthy that in this design, all RRs maintain a uniform diameter, while the separation distances between the rings and waveguides can be adjusted based on the specific functionality they serve.

Furthermore, this design operates under continuous wave conditions at a single operating frequency, denoted as ω_0_. This characteristic enables us to exert precise control over the phase and amplitude of transmitted signals by adjusting the refractive index of each component. In essence, this allows for fine-tuning the neural network’s performance [106]. Figure 7i displays a waveguide’s transmission and phase responses of side-coupled with a ring as a function of phase detuning, Δ*ϕ*. These responses are shown for both the critically coupled and over-coupled scenarios. In the case of over-coupling, indicated by the components colored in green, these responses are utilized for phase-tuning purposes. On the other hand, the nonlinear activation ring, highlighted in blue, requires critical coupling to achieve a more extensive amplitude tuning range. Figure 7j presents an illustrative example of the transmission and phase response of the coupled double ring employed as a signal-mixing component. The key parameters involved here are the ring-waveguide coupling coefficient (𝑟𝑟𝑤) at 0.85, the ring-ring coupling coefficient (𝑟𝑟𝑟) at 0.987, and the single round trip amplitude transmission (𝑎) at 1 [106].

## 4. Applications of Neuromorphic Photonics

Neuromorphic photonics represent a burgeoning interdisciplinary realm that melds the core principles of optics and neuroscience, forging the path for innovative technologies that span a diverse array of applications. A prominent use case is found in neuromorphic computing [109], harnessing the swiftness and efficiency of light to faithfully emulate the information processing capabilities intrinsic to the human brain. This paves the way for creating high-speed and energy-frugal computing systems, markedly advantageous for tasks like pattern recognition, machine learning, and intricate data analysis [109]. Moreover, the realm of neuromorphic photonics extends its reach into optical neural networks, poised to reshape data processing across various domains, including telecommunications, image recognition, and autonomous vehicles [110,111,112]. Additionally, it plays a pivotal role in elevating the performance of brain-machine interfaces, facilitating smoother interactions between humans and computers. In sum, neuromorphic photonics holds the key to propelling an array of technological domains forward, thanks to its combination of speed, efficiency, and unique capacity to replicate the computational prowess of the human brain.

### 4.1. Neuromorphic Computing Based on PICs

As we approach the limits of Moore’s law and the termination of Dennard scaling, the computing community actively seeks new technologies to sustain and enhance performance [113,114]. One such groundbreaking computing technology is neuromorphic computing. The term “neuromorphic” was first coined by Carver Mead in the late 1980s, primarily referring to mixed analog-digital implementations of brain-inspired computing at that time [115,116]. However, as this field has evolved, with substantial funding opportunities for brain-inspired computing systems like the DARPA Synapse project and the European Union’s Human Brain Project, the definition of neuromorphic has broadened to encompass a range of hardware implementations.

In this context, neuromorphic computers are defined as non-von Neumann machines, distinguished by their structural and functional inspiration drawn from the human brain. These computers consist of neurons and synapses, unlike von Neumann computers, which comprise separate CPUs and memory units, where data and instructions are stored in distinct entities. In a neuromorphic computer, both processing and memory are governed by the neurons and synapses, with programs defined by the neural network’s structure and parameters, as opposed to the explicit instruction characteristic of von Neumann computers.

**Figure 8:**
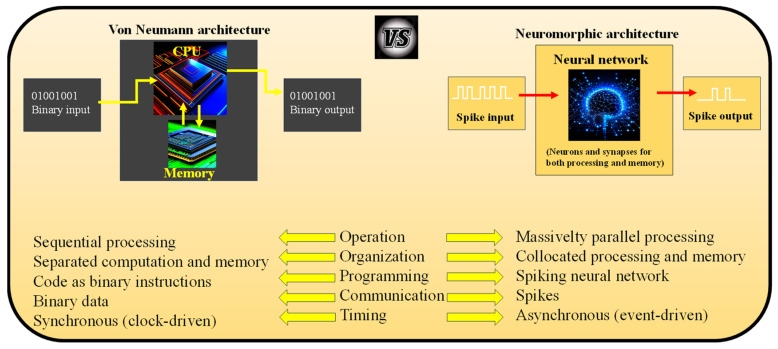
Comparison of the von Neumann architecture with the neuromorphic architecture. Inspired by [1].

Furthermore, while von Neumann computers encode information as numerical values represented in binary form, neuromorphic computers receive input as spikes. These spikes are characterized by their magnitude, timing, and shape, allowing them to encode numerical information. Converting between binary values and spikes is possible, although the precise methodology for this conversion remains an ongoing area of exploration in neuromorphic computing. These distinct characteristics underscore the exciting departure from traditional computing paradigms offered by neuromorphic computers. Neuromorphic computers exhibit fundamental operational distinctions from conventional computing architectures, as illustrated in Figure 8:

(I) Highly Parallel Operation: Neuromorphic computers inherently embrace parallelism, where all neurons and synapses can potentially operate simultaneously. However, the computations performed by these elements are relatively simple when compared to parallelized von Neumann systems.
(II) Collocated Processing and Memory: In neuromorphic hardware, there is no separation between processing and memory. Neurons and synapses are involved in processing and storage, mitigating the von Neumann bottleneck. This collocation enhances efficiency by eliminating data accesses from main memory, reducing energy consumption.
(III) Inherent Scalability: Neuromorphic computers are designed to be inherently scalable. Adding more neuromorphic chips increases the number of neurons and synapses. Multiple physical neuromorphic chips can be combined to create larger implementations, which has been successfully demonstrated in various large-scale neuromorphic hardware systems like SpiNNaker and Loihi.
(IV) Event-Driven Computation: Neuromorphic computers employ event-driven computation, meaning they only compute when data is available. This temporally sparse activity allows for highly efficient computation. Neurons and synapses operate only when spikes (data) are present, which is typically sparse within the network’s operation.
(V) Stochasticity: Neuromorphic computers can incorporate randomness, such as in the firing of neurons, allowing for noise in the system.

These characteristics collectively set neuromorphic computing apart from traditional architectures, enabling them to address the limitations of conventional von Neumann systems and pave the way for highly efficient, parallel, and scalable computing paradigms.

Neuromorphic systems, inspired by the brain’s computational processes, are pivotal for creating artificial systems that tackle problems akin to those the human brain handles. These systems promise to efficiently enable autonomous entities to understand their environment, make decisions, and execute actions effectively. Initially, early neuromorphic robots were experimental, emulating biological motion perception using ad hoc hardware and manual tuning by chip designers [117]. The field’s evolution has been significant, with readily tunable hardware accessible to non-experts through standard software tools. The development of dynamic vision sensors (event-driven) and neuromorphic computing chips with numerous neurons and synapses as computational primitives paved the way for three primary paths in neuromorphic robotics. These paths encompass visual perception for robots, connecting sensing with control, and employing spiking neural networks (SNNs) for motor control [118,119].

Simultaneously, the neurorobotics community has been working on models of perception, cognition, and behavior based on SNNs, with recent efforts to implement these models on neuromorphic platforms. Moreover, computational neuroscientists are devising learning theories that bridge deep neural networks (DNNs) with biologically-inspired spike-based learning to create spiking neural models for motor control, which could be integrated into neuromorphic hardware in the future [120,121,122]. This vibrant and diverse landscape, comprising various research communities and fields, holds the potential to usher in a transformative era. Neuromorphic sensing and computing can bolster the development of intelligent, efficient, and adaptable robots. This research is timely and essential as robots transition from highly controlled environments to scenarios where they collaborate with humans and must dynamically adapt, drawing from neural computational principles.

The notion of self-driving cars has shifted from a once-promising and near-future vision to a more complex and tempered reality. During the machine-learning boom of 2015–2020, there was significant optimism, with experts predicting widespread autonomous vehicle use by 2021 [123]. However, this optimism waned in recent years as it became clear that while making cars autonomous in plain environments like freeway driving is relatively straightforward, many real-world situations are too complex for current solutions to achieve full autonomy. These complex scenarios, often called ‘corner cases’ or ‘edge cases’, where machine learning algorithms struggle to operate correctly, have proven more common than initially anticipated.

In the industry, self-driving cars are more formally known as Advanced Driver Assistance Systems (ADAS), and the Society for Automotive Engineering’s (SAE) five-level ADAS model is commonly used to discuss autonomous driving capabilities. The current perspective on ADAS progress varies between extreme optimism and pessimism. On the one hand, some acknowledge that achieving full autonomy on a large scale is unlikely but maintain the appearance of progress to satisfy investors. On the other hand, some are in denial and genuinely believe full autonomy is imminent. Between these extremes, a consensus view among ADAS researchers is that while achieving full level 5 ADAS (full autonomy) in the next five years is unlikely, level 4 ADAS, which represents a high level of automation with some operational limitations, is both a feasible and valuable goal. A significant challenge in achieving advanced levels of ADAS is related to visual perception, particularly the difficulty of replicating human visual perception artificially. The challenge is exemplified by the fact that human drivers can deduce the intentions of a pedestrian even when they are 100 m away, which is crucial for driving at moderate to high speeds in non-freeway environments. However, replicating this capability with machine vision remains unattainable.

To illustrate, when a pedestrian is 1.5 m tall and viewed at a distance of 100 m, they appear at a tiny angle, less than 1 degree vertically and about 0.1 degrees horizontally. When captured by a video imager with a moderately wide-angle lens, necessary for forward-facing cameras in an ADAS system, this translates to approximately 3 by 16 pixels in an HD system. Even if image resolution is increased, for instance, to 10 by 85 pixels in a 4K system, the challenge remains because a higher resolution also expands the area that must be searched for the relevant pixels of interest, making the problem more complex.

Electromyography (EMG) is a neurophysiological method for recording muscle movements, primarily by detecting the electrical activity generated when a muscle contracts. EMG signals are derived from the action potentials of motor units (MUs), which consist of muscle fibers innervated by motor neuron axonal branches [124]. These signals are linearly correlated with the strength of muscle contractions and the number of activated MUs. EMG can be acquired invasively with needle electrodes or superficially using electrodes on the skin, known as surface EMG (sEMG).

EMG signals find applications in clinical and biomedical fields, particularly in myoelectric prosthetics control, where they classify muscle movements. Wearable solutions for myoelectric prosthetics control exist but require improving movement classification granularity, computational resource efficiency, and power consumption. Since EMG signals are susceptible to various forms of noise and interference, they require preprocessing, involving filtering, amplification, compression, and feature extraction in both time and frequency domains [125]. Movement classification is typically done using machine learning (ML) algorithms, which offer high accuracy but may face limitations in varied test conditions and require significant computational resources. DL techniques can improve generalization but remain computationally intensive, making them less suitable for wearable solutions [126].

Neuromorphic technologies provide a promising solution by processing EMG data with low latency and minimal power consumption, mirroring the brain’s computational principles. Compared to conventional ML approaches, neuromorphic EMG processing significantly reduces power consumption and latency, with a relatively small loss in accuracy. Some innovative approaches aim to extract motor neuron activity from EMG signals directly as spike trains [127], offering a more natural interface with muscles. However, these approaches often rely on traditional ML techniques and have yet to explore the potential of more suitable frameworks such as SNNs.

### 4.2. Neuromorphic Imaging

Neuromorphic imaging represents a cutting-edge technological approach that draws inspiration from the human brain’s intricate neural networks to advance the field of image sensing and processing. Unlike traditional digital cameras, which capture and process images using conventional methods, neuromorphic imaging systems emulate the brain’s neural architecture to acquire and analyze visual information efficiently. These systems mimic the brain’s ability to focus on relevant details while conserving computational resources quickly and selectively [128]. By leveraging neuromorphic principles, such as event-driven sensing and sparse coding, these imaging systems promise substantial advancements in low-power, high-speed image processing, making them particularly suitable for applications like robotics, AI, and autonomous vehicles, where real-time, energy-efficient visual perception is crucial. Neuromorphic imaging represents a promising frontier in the realm of computer vision and has the potential to revolutionize the way we capture and interpret visual data [129].

Taking inspiration from the remarkable capabilities of the human visual recognition system, an innovative imaging device designed to revolutionize image acquisition and data pre-processing was presented [130]. This approach involves infusing neuromorphic data processing into a curved image sensor array, mirroring how our brains process visual information. This cutting-edge curved neuromorphic image sensor array was built upon a heterostructure of MoS_2_ and poly(1,3,5-trimethyl-1,3,5-trivinyl cyclotrisiloxane). What sets this curved neuromorphic image sensor array apart is its ability to exhibit photon-triggered synaptic plasticity, a feature stemming from its quasi-linear time-dependent photocurrent generation and prolonged photocurrent decay. This unique behavior originates from the charge trapping within the MoS_2_-organic vertical stack. When coupled with a plano-convex lens, this curved neuromorphic image sensor array efficiently processes noisy optical inputs, obviating the need for redundant data storage, extensive processing, or complex optics. This innovative imaging device promises to significantly enhance the efficiency of image acquisition and recognition processes, marking a significant step forward in next-generation machine vision [130].

**Figure 9:**
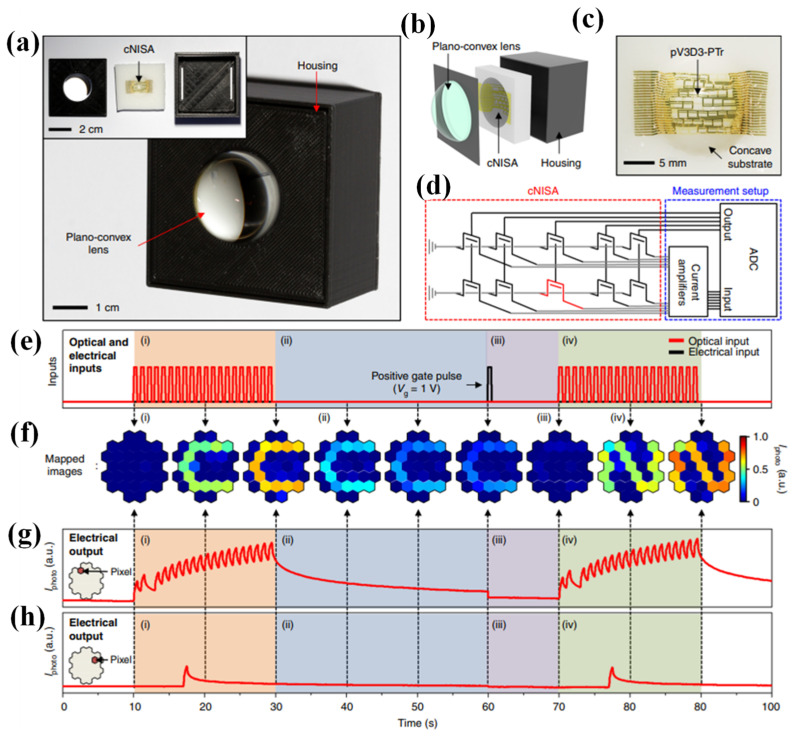
The fully integrated configuration of the curved neuromorphic imaging device is depicted in the following illustrations: (**a**) A photograph of the integrated imaging system, which comprises a plano-convex lens, cNISA, and housing. The inset provides a view of the components before they are assembled [130]. (**b**) An exploded diagram illustrating the components of the curved neuromorphic imaging device [130]. (**c**) A photograph of cNISA positioned on a concave substrate [130]. (**d**) A schematic representation of the custom-designed data acquisition system utilized for measuring the photocurrents of individual pixels in cNISA. (**e**–**h**) Demonstrations showcasing the process of obtaining a pre-processed image from a large set of noisy optical inputs. This includes the acquisition of a pre-processed C-shaped image (**i**), the gradual fading of the memorized C-shaped image (**ii**), the erasure of any residual afterimage (**iii**), and the acquisition of a pre-processed N-shaped image (**iv**), (**e**) displays the applied optical and electrical inputs, while (**f**) shows the obtained images at various time points [130], (**g**,**h**), the photocurrents recorded from specific pixels at each time point can be observed [130].

To demonstrate more intricate patterns in the imaging experiments, the size of the pixel array was increased from 9 pixels to 31 pixels, and incorporated various components into the developed integrated imaging system, as shown in Figure 9a. This integrated system comprises a plano-convex lens responsible for focusing incoming optical inputs, a component called cNISA that processes noisy optical inputs into pre-processed images, and a housing that provides support for both the lens and the pixel array (as shown in the inset of Figure 9a,b.

An ultrathin device structure with a thickness of approximately 2 μm, including encapsulations, and employing flexible materials such as graphene, MoS2, and pV3D3 created a pixel array that can be mechanically deformed [130]. A strain-releasing mesh design introduced patterns to reinforce fragile materials like Si_3_N_4_, and the array was positioned near the neutral mechanical plane, which was integrated. This design resulted in a minimal strain of less than 0.053% on the deformed array. Consequently, the array can be seamlessly integrated onto a concavely curved surface without experiencing mechanical failures, as illustrated in Figure 9c. A customized data acquisition system was developed, including current amplifiers and an analog-to-digital converter (ADC) to facilitate the photocurrent measurement from cNISA, as presented in Figure 9d. Each pixel of cNISA is connected serially to the current amplifier via an anisotropic conductive film (ACF). In Figure 9e–h, a visual demonstration of the image acquisition and pre-processing process was presented using the integrated system. Initially, the system was exposed to noisy optical inputs in the form of C-shaped images, consisting of 20 optical inputs with durations of 0.5 s and intervals of 0.5 s. More details can be found in [130].

Neuromorphic vision sensors, drawing inspiration from biological vision, employ an event-driven, frameless approach to capture abrupt changes in visual scenes. This sets them apart from conventional cameras in that they exclusively relay localized pixel-level alterations, referred to as “events,” triggered by movement in a scene as they happen [130,131]. The result is an information-rich stream of events with a remarkable latency within tens of microseconds. To be more precise, a single event comprises a tuple (t, x, y, p) consisting of x, y pixel coordinates in a 2D space, a timestamp (t) indicating when the event occurred, and a polarity (p) denoting whether the brightness change was increasing or decreasing.

**Figure 10:**
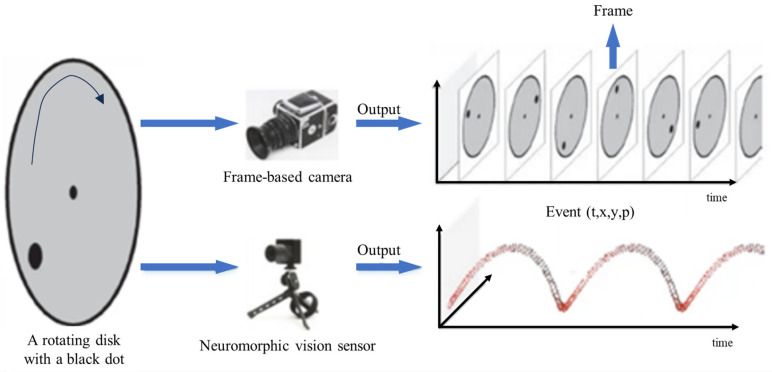
The contrast between the output generated by a neuromorphic vision sensor and a traditional frame-based camera when observing a spinning disk with a black dot. Compared to the regular frame-based camera, which transmits entire images with a consistent delay, the neuromorphic vision sensor emits events independently and without a fixed schedule, corresponding to the moments when they occur [132].

Moreover, the sparse nature of the event stream drastically reduces the demands on data storage and computational resources. In addition to its low latency and high storage efficiency, neuromorphic vision sensors offer an impressive dynamic range of 120 dB. These characteristics of neuromorphic vision sensors serve as a wellspring of inspiration for the development of entirely new intelligent transportation system designs. To provide a clearer understanding of how neuromorphic sensors function, Figure 10 offers a comparison between standard frame-based cameras and neuromorphic vision sensors.

Over the past decade, a growing focus has been on detecting and monitoring multiple vehicles in traffic environments for traffic surveillance, traffic control, and road traffic information systems. This field represents a burgeoning area of research within intelligent transport systems (ITSs) [133,134,135]. Most current vehicle tracking systems rely on video cameras [136]. Previous methods for vision-based detection and tracking of multiple vehicles can be categorized into four main approaches: frame difference and motion-based techniques [137,138], background subtraction methods [139], and feature-based methods [140,141]. Furthermore, a few datasets based on camera imagery for vehicle detection and tracking have become available in recent years, spurring advancements in the field [142,143].

Until now, all the methods for detecting and tracking multiple vehicles have relied on images captured by traditional frame-based cameras. However, these conventional cameras can encounter motion-related issues (for example, motion blur, rolling shutter) that affect their ability to detect and track high-speed vehicles effectively. Neuromorphic vision sensors have extensively used in robotics [144] and vehicles [145]. In recent years, a few relevant neuromorphic vision datasets have been released, further promoting the application of neuromorphic vision for object detection and tracking [146]. Moreover, there has been a growing trend in employing neuromorphic vision sensors for various detection and tracking tasks, including feature tracking, line tracking, and microparticle tracking [147,148].

Nevertheless, despite numerous advancements in the field, there is need for more neuromorphic datasets and associated applications for neuromorphic vision sensors in ITSs. It is worth noting that these sensors possess inherent advantages when recording high-speed motion, which could significantly enhance the detection and tracking of multiple vehicles in ITSs operating at high speeds. Consequently, applying neuromorphic vision techniques in ITS systems holds substantial potential and significance.

**Figure 11:**
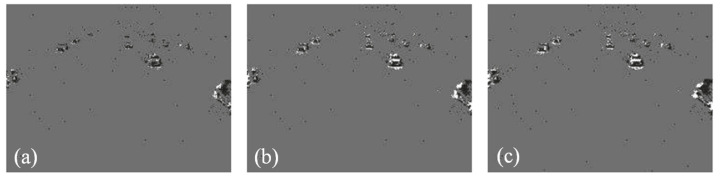
(**a**) Events gathered within a 10 ms time span [149]. (**b**) Events collected over a 20 ms time interval [149]. (**c**) Events compiled during a 30 ms time duration [149].

In [149], the pioneering neuromorphic vision-based multi-vehicle detection and tracking system for ITSs was introduced. The system’s performance was assessed using a dataset captured by a neuromorphic vision sensor installed on a highway bridge. The initial investigation involves multi-vehicle tracking through clustering, employing three classical clustering methods and four tracking techniques. The experimental findings validate that leveraging the advantages of low latency and a sparse event stream allows seamless integration of an online tracking-by-clustering system that operates at a high frame rate, surpassing the real-time capabilities of traditional frame-based cameras. Moreover, if accuracy is the foremost concern, the tracking tasks can be robustly executed at a relatively high rate using various algorithm combinations. The dataset and evaluation methods are available, thus establishing the first neuromorphic benchmark in ITS. The event data is contemplated as pure two-dimensional point data. The clustering techniques are employed to generate object proposals. The event data collected over different time intervals (10 ms, 20 ms, and 30 ms) are aggregated and visualized in Figure 11a–c. Clusters of event data correspond to moving vehicles, while the noise events around these clusters primarily result from environmental fluctuations and sensor noise.

A background activity filtering step was implemented to refine the object hypotheses to eliminate noise from the events. This step involves examining each event and checking whether any of its eight neighbouring pixels (both vertically and horizontally) have recorded an event within the last “us Time” microseconds. If not, the event in question is categorized as noise and removed. In principle, the determination of whether a new event is considered a “signal” or “noise” hinges on the presence of a neighbouring event occurring within a specified time interval (us Time). Applying the activity filter helped to improve the detection quality [150]. 

### 4.3. Image Processing by Neuromorphic Structures

For the initial evaluation and validation of an optical neural network, many authors prefer to choose a classification, clustering, or image recognition task based on the well-known MNIST dataset. Still, they need to include the applicability of their architectures to other tasks solved by neural networks. Globally, existing photonic structures for the realization of neural network operation, depending on the type of source in the circuit, are divided into two subspecies: coherent and incoherent architectures. The first variant implies that the input light is used in an array of beam splitters and phase shifters to perform matrix computation operations using interference between different paths, i.e., in this variant, it is possible to use a single laser source but with sufficient power [151]. Such an architecture is often built on Mach–Zehnder interferometers [50,152]. An incoherent architecture uses multiple sources operating at different wavelengths or a single source but uses WDM techniques [151,153].

**Figure 12:**
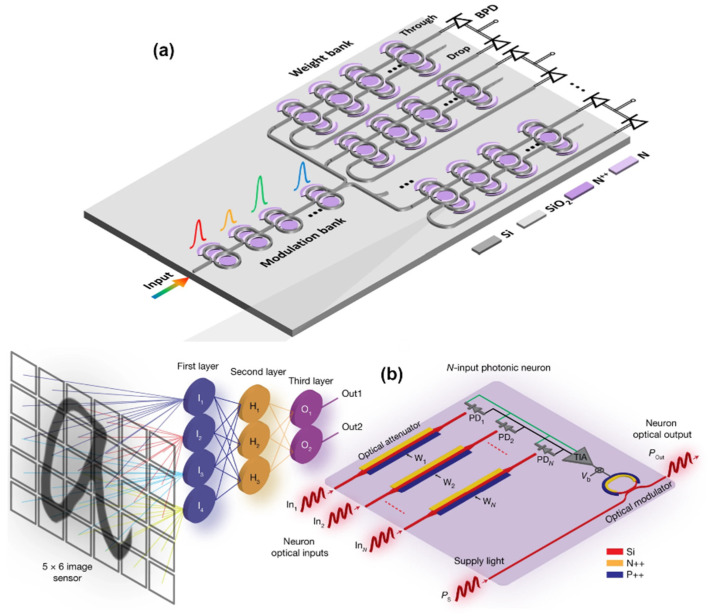
(**a**) Schematic diagram of the developed photonic circuit using IM-MRR technology [151]; (**b**) structural diagram of the photonic chip including all elements to realize the operation and illustration of the process of splitting the original image into pixel components and feeding data to each layer of the network [154].

Each architecture has pros and cons, different application areas, and element bases applicable to the operation scheme. For example, ref. [151] presented a photonic circuit that uses a multi-WDM architecture. The circuit introduces interferometric modulators based on microring modulators (MRMs) that contain photoconductive heaters that play the role of index modulation components and are used for tracking resonant peaks (Figure 12a). The use of intensity modulation and MRM-based interferometric modulators represents a novel approach that improves the efficiency and flexibility of neural networks. These innovative techniques increase the dimensionality of the tensor kernel and provide more accurate tracking of resonant peaks, leading to improved performance and accuracy of calculations in neural networks. The application of the developed modulation scheme by intensity allowed channel compaction by wavelength 17 times higher than that of traditional analogs with modulation by wavelength, which allowed the use of 578 channels with permissible power losses of 3 dB. Simulation results using the MNIST handwritten digit recognition dataset show accuracy up to 96.76%, with a mean square error (MSE) of 3.09 × 10^−3^. The results also show that using IM-MRM (intensity-modulation-based microring modulators) for optical data processing avoids inter-channel crosstalk.

A new structure is presented in [154], a photonic deep neural network (PDNN) for subnanosecond image classification, which is unique for neuromorphic realization. Its originality lies in the authors realizing the whole data processing procedure, starting from image input and ending with computation of the NN result on a single photon chip, which is a challenging task. In particular, the main challenges are the image translation into an optical domain for further processing on the photon chip and realization of the nonlinear activation function. These problems were solved in the following ways: a laser at a wavelength of 1532 nm is used for image formation, which illuminates A transparent film with letters printed on it after passing through a collimator, then the light hits a grid with grating couplers, which act as pixels (5 × 6 pixel classification matrix); an MRM with the addition of a PN junction was used as an element of the ReLU activation function. The weighting coefficients were realized by creating PIN attenuators on the direct input waveguides. The structure of the photonic neural network is shown in Figure 12b. Significant emphasis in the study was focused on achieving high-speed image classification. As can be seen, awe-inspiring results (570 ps) were obtained, outperforming existing electronic and other optical structures. Classification accuracy reached 93.8%. The presented architecture has great potential and significant advantages, including the absence of digitization processing steps and the need for memory modules, which makes it possible to achieve such results in image processing speed.

Another promising approach to realizing the operation of optical neural networks is the use of VCSELs (Vertical-Cavity Surface-Emitting Laser) [112,155]. In such solutions, VCSEL represents an artificial optical neuron that integrates and processes information from multiple inputs. The paper [112] introduces an artificial optical VCSEL neuron as a neural circuit. Like a biological neuron, this neuron integrates multiple inputs and generates a spike when the input exceeds a certain threshold. The circuit consists of various components, including an arbitrary waveform generator (AWG) for generating image input data, a Mach–Zehnder intensity modulator (MZ) to encode the data into the light emitted by a tunable laser (TL), and fiber-optic components such as optical isolators (ISO), variable optical attenuators (VOA), polarization controllers (PCs), and circulators (CIRC) to direct the laser light into the VCSEL neuron. The response of the VCSEL neuron is then analyzed using a fast real-time oscilloscope (OSC) after detection with a photodiode (PD). To process the image input data, it is converted into binary matrices and operated on by a 2 × 2 kernel operator. The resulting data is time-multiplexed to generate a return-to-zero (RZ) image input, where each pixel has a configurable duration. The VCSEL neuron exhibits fast optical spikes (100 ps-long) when it detects desired image features.

Furthermore, it is combined with a software-implemented spiking neural network to perform complex image classification tasks. The system operates at a high speed by utilizing 100 ps-long inputs and is designed to be compatible with hardware, relying on a single VCSEL device and time division multiplexing. It successfully demonstrated the ability to detect edge features in images using an all-optical neuromorphic approach with a VCSEL neuron, showcasing its resilience to image noise. Moreover, the system processed 5000 images from the MNIST handwritten digit database, achieving an impressive mean image classification accuracy of 96.1% when combined with a software-implemented SNN. The theoretical demonstration of the VCSEL neuron’s capability to handle larger dimension kernels, such as 3 × 3, for more complex image feature extraction further highlights its potential. Artificial spiking VCSEL neurons hold great promise for future high-speed, low-energy, and hardware-friendly neuromorphic photonic platforms dedicated to image processing, offering a fast telecom-compatible spiking representation.

Currently, all existing implementations of neuromorphic networks require electronic components. They play an essential role in controlling and tuning neural networks, acting as weighting factors or assisting elements for activation functions, and in the pre-and post-processing of results. This indicates that optical neural networks are currently not intended to replace digital solutions but rather to improve their performance and data processing efficiency.

## 5. Materials Used for Implementation of Photonic Neuromorphic Computing

**Figure 13:**
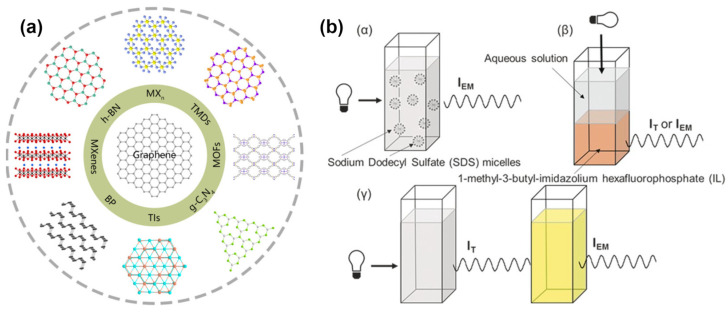
(**a**) Diagram of typical structure of 2D layered materials [157]; (**b**) the communication architectures: α architecture, both the transmitter and the receiver are located within the same cuvette and phase, potentially with one component protected by micelles. In the β architecture, the transmitter and receiver are situated in the same cuvette but in two immiscible phases (water-ionic liquid). In the γ architecture, the transmitter(s) and receiver(s) are placed in separate cuvettes. The networks have been achieved by combining or enhancing the α, β, and γ architectures through hybridization or upgrades [162].

The development of neuromorphic photonics also leads to the development of specific devices and components for optical neural networks. Select materials have advantages for some tasks and functions but may be unsuitable for other components. Therefore, the study of the issue of materials used to realize neuromorphic computing is best done for individual components. For example, two-dimensional (2D) organic materials such as perylene-3,4,9,10-tetracarboxylic dianhydride (PTCDA) including 2D transition metal dichalcogenides (TMDCs) such as molybdenum disulfide (MoS_2_) and tungsten selenide (WSe_2_) have demonstrated promise for the development of complex neuromorphic networks. Thus, in [156] a multifunctional transistor of artificial neural synapse based on a fully 2D inorganic/organic (hybrid) MoS_2_/PTCDA heterostructure on SiO_2_/Si substrate with neuromorphic STP (short-term plasticity) and LTP (long-term plasticity) functions with both electrical and optical modulation and efficient gate tunability was demonstrated for the first time. Due to their remarkable nonlinear optical properties, layered materials have started to find many applications in the field of nonlinear photonics. The paper [157] provides an excellent detailed analysis of existing synthesized two-dimensional layered materials with synthesis methods, nonlinear optical properties and directions for integration of layered materials into photonics devices (Figure 13a illustrating the diagram of typical structure of 2D layered materials). The choice of material and platform in general is driven by the required optical and electrical properties of the components and the tasks they have to perform in the network.

The function of synapses in neuromorphic networks is also dependent on electrical stimulation. Therefore, in [158] the authors undertook the challenge of creating fully photon modulated synapses that would be able to emulate both excitatory and inhibitory synaptic behavior. To achieve this target, the study employed ZnO films and PbS QT (quantum dot) using a glass/indium tin oxide (ITO)/ZnO/PbS/ZnO/Al structure. This choice is based on the ability of ZnO to absorb short wavelength light and PbS to absorb long wavelength light. The conductivity of the materials changes gradually due to the change of oxygen vacancies under photon stimuli, and excitation and inhibition occur when exposed to long- and short-wavelength photons, respectively. Another unique device applicable for solving synaptic problems for neuromorphic structures is the photonic neurotransistor. In [159] the authors introduce a photonic neurotransistor based on metal-chalcogenide and metal-oxide materials, capable of emulating synaptic responses and neural computation. These neurotransistors differ from conventional transistors as they are specifically designed to mimic synaptic reactions and neural computation. The heterogeneous semiconductor channel structure consists of a broadband photoactive layer of CdS stacked on top of a visible light-insensitive ZTO layer. This configuration enables the neurotransistors to receive multispectral pulses, integrate the received signals, and perform computations. The multispectral gate triggers and their corresponding synaptic responses are emulated using a broadband absorbing heterogeneous MC/MO semiconductor structure and its defect heterointerface, which can be finely adjusted by modifying the photospectrum of the applied spikes and controlling the interfacial traps between them, respectively.

In general, there are many more directions for the development of photonic or optoelectronic synapses for the realization of neuromorphic networks with detailed justifications of the choice of certain materials for their embodiment [160,161]. In [161] an extensive analysis of photonic synapses based on various potential materials (metal oxides, perovskites, low-dimensional materials, organic materials, and phase transition materials) was performed. The review presents recent advances in photonic synapses and their applications in neuromorphic systems. Photonic synapses offer unique advantages over synapses investigated using an electrical stimulus. They provide ultra-high propagation speed, high bandwidth, and low crosstalk, which contributes to increased computational speed and enables optical wireless communication.

Another unique area of neuromorphic structure development is wetware devices (i.e., in wet solutions) [162,163,164]. “Wetware” software contributes important advantages to the efficiency of neuromorphic networks due to the chemical compounds and reactions that occurr in the process which are similar to real information processing in the human brain. The authors in [162] demonstrate that liquid implementation offers several key advantages over solid-state counterparts. These advantages include the ability to facilitate long-range interaction phenomena mediated by diffusive chemicals, similar to multicellular systems with complex chemical signaling. These long-range coupling effects enhance the computational capabilities of the entire system by generating collective oscillations and waves. Additionally, liquid implementation enables information encoding using UV/visible radiation, which significantly increases the speed of message propagation. Furthermore, the network has the capability to communicate with a diverse range of chemical compounds. This study utilized oscillatory reactions (Osc) and excitably photochromic or luminescent species (Exc) as models of artificial neurons, and investigated their interaction through UV/visible radiation. The communication architectures (α, β, γ) are illustrated in Figure 13b. Here is another unique chemical neurocomputer architecture realized in [164] using the Belousov–Zhabotinsky reaction due to its ability to operate continuously under batch conditions, generating numerous spikes with a periodicity of approximately one minute. The network composed of pulse-coupled chemical micro-oscillators and excitable micro-cells, which includes components such as the central pattern generator, readers, antenna, and decision-making unit, demonstrated intelligent responsiveness to external signals. It automatically transitioned from its current dynamic mode to a new mode, resembling the induced dynamic mode of the antenna. This behavior indicates the network’s capacity for intelligent adaptive behavior, highlighting the potential of the chemical “neurocomputer”.

At the moment, the study of photonic synapses is still in the early stages of development. However, by using new materials, it has been possible to successfully model basic synaptic behaviors such as EPSC (excitatory postsynaptic current), PPF (paired-pulse facilitation), STP, LTP and the transition from STP to LTP. This opens new possibilities for the development and application of photonic synapses in future neuromorphic systems [161].

## 6. Current Challenges

The burgeoning field of neuromorphic photonics stands at the fascinating crossroads of neuroscience and photonics. It possesses the extraordinary ability to meld the information-processing capabilities of neuroscience with non-von Neumann architectures and the unique attributes of photonics. These attributes include virtually boundless bandwidth, exceptional speed, remarkable power efficiency, multidimensional multiplexing capabilities, and a fundamental immunity to electromagnetic interference. What makes neuromorphic photonics even more compelling is their compatibility with mature microelectronics in the form of Complementary Metal-oxide-semiconductor (CMOS) integration, making them a promising option for future neuromorphic computing hardware.

Neuromorphic photonics primarily employs low-loss waveguides, high-efficiency couplers, high-speed modulators, and high-sensitivity photodetectors to create high-performance, energy-efficient computing architectures. The primary energy consumption occurs during input preparation, weight adjustment, nonlinearity activation, and output detection. In integrated nonvolatile memory cases, energy consumption becomes minimal for weight maintenance or phase control once the PCM elements are trained. Theoretically, matrix multiplication can be performed passively at the speed of light. However, achieving an efficient integrated neural network still presents several scientific and technological challenges.

First and foremost, building a complete neuromorphic photonic computing ecosystem, where light sources, passive and active components, and transistors collaborate, is a formidable task. There is currently no single commercial fabrication platform capable of simultaneously achieving all these components on a single die. Existing on-chip optical light or gain sources require complex fabrication processes, such as cointegration of III–V materials or direct epitaxy, which may not meet commercial standards in terms of reliability. Integrating photonic systems with transistors and low-power CMOS controllers to enable electrical control, feedback, and stabilization is crucial for robust Photonic Neural Networks (PNNs). These challenges stem from using different photonic materials with mostly incompatible foundry processes.

Secondly, achieving low power consumption and nonvolatile photonic storage and weighting is essential to enable neurosynaptic functions. While neural nonlinearities have been demonstrated on mainstream platforms using various techniques, energy efficiency and fast switching with new integrable materials remain areas of opportunity.

Third, there is a growing demand for fully reconfigurable integrated PNNs capable of performing complete ANN operations. Silicon photonics is emerging as an ideal platform for integrating these components, offering foundry compatibility, compact devices, and cost-effectiveness. Ongoing developments in silicon photonics devices make constructing high-performance integrated silicon PNNs with highly functional optical components feasible. The emergence of multi-project wafer (MPW) services in silicon photonics from commercial electronics foundries and research institutions further fuels the potential of fully integrated PNNs.

Lastly, to harness the combined power of photonics and neuroscience and translate it into real-world applications, significant strides are needed to bridge current neural network algorithms with the physical response of PNNs. The literature currently presents only a few proof-of-concept PNNs with limited control units and neural algorithms for basic recognition scenarios. The ideal scenario involves neural network programming tools compatible with electronic AI, which will soon facilitate the direct reconfiguration of large-scale neuromorphic photonic processors. Ultimately, self-contained PNNs must contend with high-performance computers, requiring robustness in various environments, universal algorithms, and seamless interfaces with electrical processors. These aspects promise to be key focal points in the evolving landscape of neuromorphic photonics.

## 7. Concluding Remarks

Neuromorphic photonics represents an emerging and innovative frontier at the confluence of photonics and neuromorphic engineering. The primary objective of this field is to develop accelerated processors that harness the remarkable information processing capacity of neuromorphic architectures while capitalizing on the exceptional speed and bandwidth offered by photonics. This pursuit is driven by the ever-widening chasm between existing computing capabilities and the escalating computing demands, mainly stemming from the limitations inherent in conventional microelectronic processors, particularly in the high-performance computing (HPC) domain. This challenge becomes increasingly evident in applications involving intricate systems, managing extensive volumes of data, and real-time data processing. These tasks are consistently hindered by the computational bottleneck posed by multiply-accumulate (MAC) operations. On the other hand, the analysis shows that it is too early to talk about a completely optical computer. Most works describe optical computing units designed to solve specific problems, for example, data processing using optical neural networks or matrix multiplexing. This brings neuromorphic photonics devices closer to ASICs (application-specific integrated circuits), so the most probable application of these optical devices now is joint processing of computing units with traditional electronic processors.

## Data Availability

Not applicable.

## Abbreviations

Photonic neural networks = PNNs; Machine learning = ML; Microring resonator = MRR; Ring resonator = RR; Wavelength selective switch = WSS; Convolutional neural networks = CNNs; Temporal convolutional operations = TCOs; Neural network = NN; Artificial intelligence = AI; Complementary Metal-Oxide-Semiconductor = CMOS; Photonic Neural Networks = PNN.
